# What Makes the Harderian Gland Transcriptome Different From Other Chicken Immune Tissues? A Gene Expression Comparative Analysis

**DOI:** 10.3389/fphys.2018.00492

**Published:** 2018-05-08

**Authors:** Melissa S. Deist, Susan J. Lamont

**Affiliations:** Department of Animal Science, Iowa State University, Ames, IA, United States

**Keywords:** Harderian gland, relative expression analysis, bursa of fabricius, thymus gland, spleen, chicken

## Abstract

The Harderian gland is a sparsely characterized immune tissue known to play an important role in local immunity. The function of the Harderian gland, however, is not clearly defined. Measuring the expression of all genes using RNA-seq enables the identification of genes, pathways, or networks of interest. Our relative RNA-seq expression analysis compared the chicken Harderian gland transcriptome to other important primary and secondary immune tissues including the bursa of Fabricius, thymus, and spleen of non-challenged birds. A total of 2,386 transcripts were identified as highly expressed in the Harderian gland. Gene set enrichment showed the importance of G-protein coupled receptor signaling and several immune pathways. Among the genes highly expressed in the Harderian gland were 48 miRNAs, a category of genetic elements involved in regulation of gene expression. Several identified miRNAs have immune related functions. This analysis gives insight to the unique immune processes inherent in the Harderian gland.

## Introduction

Avian species have many unique immunological features compared to mammals with whom they last shared a common ancestor over 310 million years ago (Hedges, 2002). In birds the spleen is the largest lymphoid tissue, but is only able to encounter antigens that circulate through the blood because unlike mammals, birds lack a lymphatic system (Oláh et al., 2013). T cell development is similar in mammals and birds, but chickens have more γδ T cells than humans (Smith and Göbel, 2013). The thymus is a primary immune tissue where T cell development, differentiation, and maturation occurs.

Humans completely lack the bursa of Fabricius and only have a rudimentary Harderian gland, whereas these two tissues play very important roles in the chicken immune system. The bursa is a unique primary immune organ found in birds that plays a critical role in the immune response. B cell development, proliferation, and diversification occurs in the bursa, where B cells also undergo immunoglobulin rearrangement to create B cell receptors and mature B cells (Glick et al., 1956). The Harderian gland is located behind the eyes of the chicken and its function is not clearly defined, but includes the lubrication of the nictitating membrane (Bang and Bang, 1968). It is a relatively small tissue; in adult chickens the average weight was found to be 84.4 mg (Wight et al., 1971). The Harderian gland is known to contain many B cells. The majority of cells within the Harderian gland react to anti-chicken bursa cell serum (Albini and Wick, 1974). Lymphocytes from the bursa migrate to the Harderian gland prior to hatch and may not be involved in systemic immunity (Mueller et al., 1971; Baba et al., 1988). Also, terminal B cell maturation may occur in the Harderian gland (Manisikka et al., 1989). The Harderian gland is also home to large numbers of T cells. Equal numbers of CD3+ and Bu-1+ cells were found in the Harderian gland of both control and vaccinated chicks, and there were twice as many CD4+ than CD8+ cells in unvaccinated chicks as measured by immunostaining using monoclonal antibodies (Russell et al., 1997). The B and T cells of the Harderian gland play an important role in local immunity (Manisikka et al., 1989; Maslak, 1994). The bursa, thymus, spleen, and Harderian gland are among the most important immune tissues in the chicken.

Previously, these four immune tissues were compared directly via immunohistochemistry staining. In ducks, induction of CD8+ cell immunity within the spleen and thymus was stronger after challenge with an attenuated strain of hepatitis A, whereas in the bursa and Harderian gland CD8+ cell immunity was induced more strongly after challenge with the virulent strain (Ou et al., 2017). These tissues respond differently to antigen. In unstimulated chickens, μ Heavy chain and λ Light chain mRNA were expressed higher in the Harderian gland than the bursa, spleen, and thymus (Manisikka et al., 1989). Studying the transcriptome of these tissues elucidates the mechanisms utilized in response to pathogens. Until recently, the Harderian gland transcriptome had never been analyzed (Deist et al., 2018).

Unlike the Harderian gland, the transcriptomes of the bursa, spleen, and thymus tissues are well-characterized. Transcriptome analysis of the bursa revealed BCR receptor signaling and cytokine–cytokine receptor interaction pathways were impacted by avian pathogenic *E. coli* (APEC) (Sun et al., 2015), apoptosis of IgM+ cells, infiltration of macrophages, and increased expression of pro-inflammatory genes were seen after infection with velogenic Newcastle disease virus (NDV) (Kristeen-Teo et al., 2017), and defense response to virus, positive regulation of T cell-mediated cytotoxicity, and *IFN*-γ production pathways were impacted by infectious bursal disease virus (IBDV) infection (Ou et al., 2017).

The spleen transcriptome responded to a combined heat stress and LPS treatment by altering the expression of genes within the Hepatic Fibrosis/Hepatic Stellate Cell Activation and Macrophages pathway in two distinct genetic lines (Van Goor et al., 2017). In response to APEC infection, broiler splenic gene expression was predicted to affect the Jak-STAT and cytokine–cytokine receptor signaling pathway (Sandford et al., 2011), and the spleen responded to NDV challenge by activating interferon-stimulated genes (Zhang et al., 2018).

Compared to the bursa and spleen, there have been relatively few RNA-seq studies conducted on the chicken thymus. The thymus transcriptome responded to APEC by impacting the TLR signaling pathway, lysosome pathway, CAMs, and TCR signaling pathway (Sun et al., 2016). Another study showed thymus atrophy and its possible relationship with the expression of immune genes after challenge with LPS and *Salmonella* (Huang et al., 2016). In response to heat stress and an LPS challenge in the thymus transcriptome, ILK Signaling, Integrin Signaling, and cell proliferation pathways were all impacted (Lamont, personal communication).

Within each immune tissue, pathogen, strain, dose, time, genetic line, and more, greatly impact gene expression. Under basal conditions it is unclear how these tissues’ transcriptomes compare, especially how they compare to the Harderian gland. A relative expression analysis of these fundamental immune tissues will help to better characterize the Harderian gland by identifying genes highly expressed (relative expression value greater than 2 SD from the mean) in this tissue relative to the bursa, thymus, and spleen. We assume the genes highly expressed in the Harderian gland are either related to tissue-specific non-immune function of the gland, or related to the unique immune function of this tissue in contrast to the other immune tissues studied. We hypothesize that the Harderian gland has mechanisms of defense that can be triggered rapidly because of its role in local immunity compared to the other more systemic immune tissues, and that the functional analysis of the genes highly expressed in the Harderian gland compared to the bursa, thymus, and spleen may elucidate these mechanisms.

## Materials and Methods

### Sample Descriptions and Processing

The Fayoumis (Line M 15.2) from the Iowa State University Poultry Farm (Ames, IA, United States) have been maintained as an inbred line since 1954 resulting in an inbreeding coefficient of 99.95% (Fleming et al., 2016). All publically available RNA-seq data comes from the non-challenged Fayoumi controls from either a NDV challenge experiment (Experiment 1) (Deist et al., 2018; Zhang et al., 2018) or a combined heat stress and LPS experiment (Experiment 2) (Van Goor et al., 2017) (**Table 1**). In both experiments the Fayoumis were raised in floor pens with wood chips and *ad libitum* access to food and water. Although performed in separate batches, all tissues were collected and placed into RNAlater solution (Thermo Fisher Scientific, Waltham, MA, United States) for short-term storage, tissues were homogenized using mechanical disruption, RNA was isolated using an RNAqueous kit (Thermo Fisher Scientific, Waltham, MA, United States), DNAse treated with the DNA-free kit (Thermo Fisher Scientific, Waltham, MA, United States), and assessed for quality (RQN or RIN > 8). All samples underwent the same protocols to generate the cDNA libraries (TruSeq RNA sample preparation guide (v2; Illumina, San Diego, CA, United States), and were sequenced on the same HiSeq2500 machine to generate 100 bp single-end reads at the Iowa State University DNA Facility (Ames, IA, United States) (**Table 1**). Spleen samples were collected in both Experiments 1 and 2. From Experiment 1 at ages 23 and 27 days, three of the four spleen and Harderian gland tissue samples were from the same individuals. No spleen samples from Experiment 1 were analyzed at 31 days of age. The spleen, thymus, and bursa samples from Experiment 2 came from the same four individuals (**Table 1**).

**Table 1:** Sample information.

Tissue	Age (days)	Number of birds	Male:Female	Accession^c^
Harderian gland^a^	23	4	2:2	E-MTAB-6038
Harderian gland^a^	27	4	3:1	E-MTAB-6038
Harderian gland^a^	31	4	2:2	E-MTAB-6038
Spleen^a^	23	4	2:2	E-MTAB-5851
Spleen^a^	27	4	3:1	E-MTAB-5851
Spleen^b^	22	4	1:3	GSE85434 (GEO)
Thymus^b^	22	4	1:3	E-MTAB-6290
Bursa^b^	22	4	1:3	E-MTAB-6289


*^*a*^Fayoumi controls from Experiment 1. ^*b*^Fayoumi controls from Experiment 2. ^*c*^Data sets available from ArrayExpress (https://www.ebi.ac.uk/arrayexpress/) or GEO (https://www.ncbi.nlm.nih.gov/geo).*

The RNA-seq data underwent a standard pipeline previously described (Deist et al., 2017) and was mapped to the Gallus_gallus-5.0 (GCA_000002315.3) reference genome using TopHat2 (Kim et al., 2013). The number of reads mapped to each transcript was counted using HTSeq (Anders et al., 2015). All transcripts with less than four counts across all samples were removed resulting in 18,123 of 38,118 usable transcripts.

### Calculating Relative Expression Values

The protocol for calculating relative expression values was previously described (Bailey et al., 2009; Pritchett et al., 2017). Pritchett et al. (2017) used the Fragments Per Kilobase of transcript per Million mapped reads (FPKM) normalization method to normalize their counts. The current study has adapted the method using count data normalized with the variance stabilizing transformation in DESeq2 (fittype = mean; blind = true) (Love et al., 2014). A constant (2.32) was added to all normalized counts to make all values positive. The following formula was used to calculate the relative expression values (rEx).

$$rEx=\log_{2}\left(\frac{\text{Maximum normalized counts for each transcript in the Harderian gland}}{\text{Median normalized count for each transcript in other immune tissues}}\right)$$

A transcript’s rEx value was considered significant if it was more than two standard deviations from the mean. The same significance threshold was used previously (Pritchett et al., 2017). Comparing the maximum value to the median emphasizes the identification of transcripts highly expressed in the Harderian gland. Although this method may be sensitive to outliers, the standard deviation of individuals’ normalized counts within each transcript in the Harderian gland was on average 1.05 (maximum SD = 5.07, minimum = 2.26E-19). The individual sample from which the maximum normalized count value was obtained was well-represented across all samples. Each Harderian gland sample contributed a maximum normalized count value for at least 1,324 transcripts and at most 3,859 transcripts.

### Analysis of Relative Expression Data

For data visualization, pcaExplorer (Marini, 2016) PCA plots were generated using dds and vst normalization from DESeq2 (Love et al., 2014) accounting for tissue and individual. The top 1000 most variant transcripts were used to calculate the principal components.

Transcripts highly expressed in the Harderian gland were further analyzed using Panther (Mi et al., 2017), Ingenuity Pathway Analysis (IPA; Qiagen, Redwood City, CA, United States), and STRING (Szklarczyk et al., 2017). These transcripts were converted to their associated gene name using BioMart on Ensembl (version 89) and input into Panther. Panther recognized 757 of the 992 input genes for an overrepresentation test using the GO biological process complete annotation set and the *Gallus gallus* reference list with a Bonferroni correction for multiple testing. Ensembl transcript identifiers (IDs) and the relative expression values for the transcripts highly expressed in the Harderian gland were used as input to IPA. Of the 2,386 transcripts, 942 were mapped (identified) by IPA and used for analysis. Several canonical pathways were identified as significant, and those pathways with *p*-values less than 0.05 and included more than five genes have been reported. Associated gene names of transcripts highly expressed in the Harderian gland were input into STRING and used to generate a network. A high confidence (0.700) was used, all unconnected nodes were removed, and MCL clustering (inflation parameter = 3) was performed. A total of 836 nodes and 669 edges were included.

## Results

### Principal Component Analysis

**FIGURE 1:**
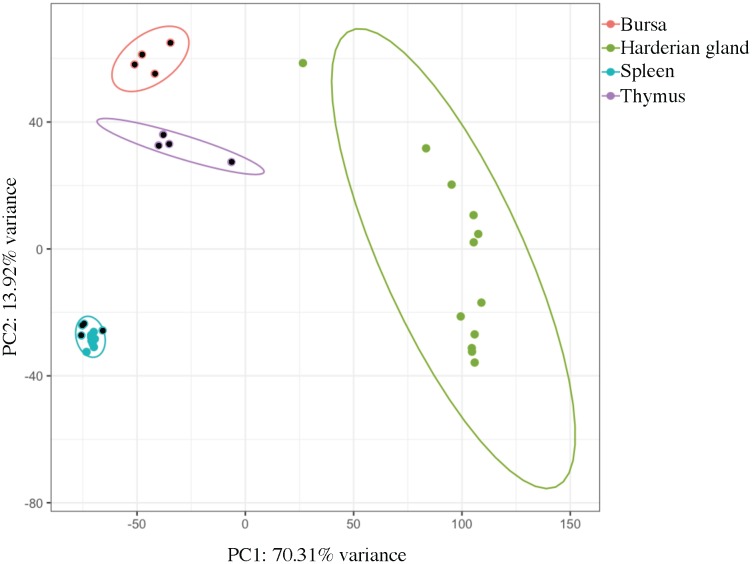
Principal component analysis shows clear clustering by tissue type. Each dot represents a tissue from an individual chicken. Principal component 1 (PC1) separated the Harderian gland samples (green) from the other immune tissues. Principal component 2 (PC2) separated the Bursa (pink), Thymus (purple), and Spleen (blue). Ellipses were drawn with a 95% confidence. Dots with a black center were from Experiment 2. This plot was generated using pcaExplorer.

Samples clustered very tightly by tissue (**Figure 1**). Bursa, spleen, and thymus samples from Experiment 2 came from the same four birds, and there was also overlap between the birds that contributed the Harderian gland and spleen samples from Experiment 1 (**Table 1**). However, no clustering by individual bird was observed. Also, samples did not cluster by age or experiment. The large first principal component (70.3%) separated the Harderian gland from the other three tissues, showing the distinctiveness of the Harderian gland transcriptome. The second principal component separated the bursa, thymus, and spleen (PC2 = 13.9%). The two primary immune tissues, bursa and thymus, clustered more closely than the spleen (**Figure 1**). The spleen samples clustered very tightly together within group even though they were different ages and from different experiments (**Figure 1** and **Table 1**). Six of the ten top loading genes for principal component 1 were also identified as highly expressed in the Harderian gland and included: *MYL1*, *MYL2*, *CKMT2*, and *TMEM182*. A list of the top and bottom loading genes was included in **Supplementary Data Sheet S1**.

### Relative Expression Analysis

**FIGURE 2:**
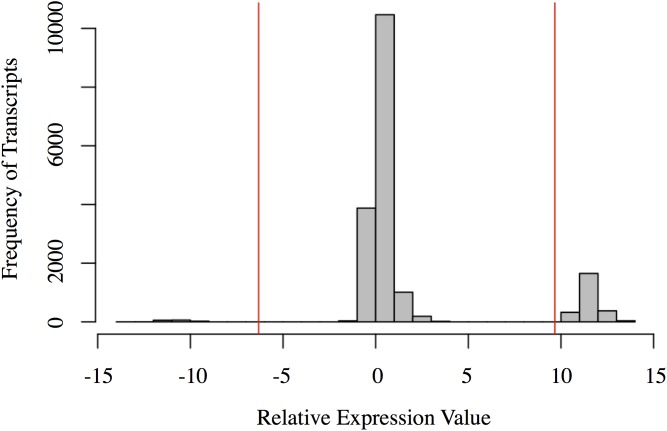
Histogram of the relative expression values for each transcript. Each bar represents the number of transcripts with a relative expression value within that range (x-axis). The red vertical lines represent two standard deviations below the mean (left) and above the mean (right). Transcripts to the left of the left red line are highly expressed in the other immune organs (spleen, bursa, and thymus). Transcripts to the right of the right red line are highly expressed in the Harderian gland. Figure generated in R.

The relative expression values followed a tri-modal distribution (mean = 1.676; *SD* = 3.997) (**Figure 2**). Gaps in the distribution may be related to the distinct clustering of the Harderian gland and other immune tissues (PC1 = 70.3%; **Figure 1**). A total of 143 transcripts were highly expressed in the non-Harderian immune tissues, whereas 2,386 transcripts were highly expressed in the Harderian gland. The 2,386 transcripts were input into Ingenuity Pathway Analysis (IPA; Qiagen, Redwood City, CA, United States) and of the 936 identified by IPA, 96 were classified as transcription regulators and 4 as translation regulators. Of the 143 transcripts highly expressed in the other immune tissues only 53 had an associated gene name. No significant GO terms were identified. Some immune genes of interest in the 143 transcripts included *C1QL3*, *C8A*, and *TLX1*. For a complete list of the 143 transcripts see **Supplementary Data Sheet S2**. The relative expression analysis was more stringent than a differential expression analysis in which 99% of the transcripts were differentially expressed (false discovery rate < 0.05; data not shown).

### Gene Set Enrichment Analyses

GO term analysis, pathway analysis, and network analysis were applied to the genes highly expressed in the Harderian gland on the assumption that these genes are the main drivers of functions that differentiate the Harderian gland from the other immune tissues. A cell type enrichment analysis (Shoemaker et al., 2012) showed no significant enrichment of any cell type based on these genes (data not shown); therefore, differences in expression levels among these tissues were likely not due to large differences in cell-type composition.

**FIGURE 3:**
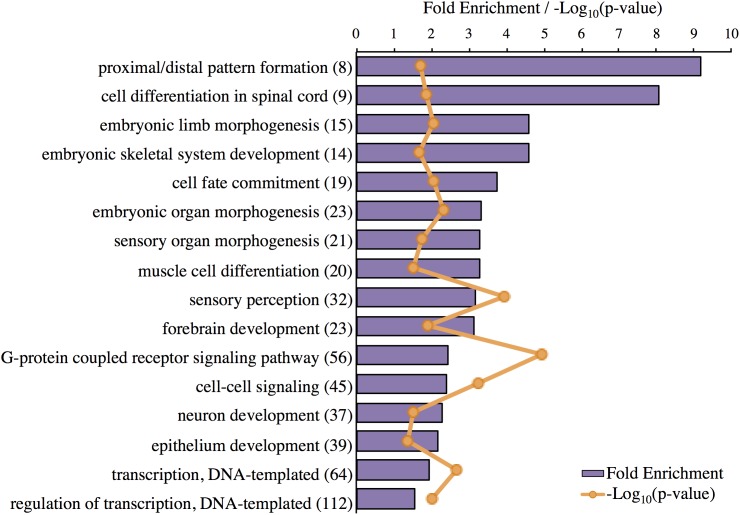
Significant GO terms associated with genes highly expressed in the Harderian gland. Transcripts highly expressed in the Harderian gland were converted to their corresponding gene ID using Ensembl (766 total) and input into Panther for statistical overrepresentation analysis using GO biological process (757 genes recognized and used for the analysis). The fold enrichment (purple bar) was calculated as the number of observed input genes divided by the number of expected genes based on the number of genes in the chicken genome. The number of genes associated with each GO term is listed to the right of the GO term in parenthesis. The Bonferroni correction for multiple testing was used to adjust *p*-values. The -Log_10_(*p*-value) for each GO term is represented by orange markers.

The top-layer GO terms identified by Panther for the genes highly expressed in the Harderian gland are shown (**Figure 3**). Most GO terms were related to development and morphogenesis. The most significant GO term was G-protein coupled receptor signaling (**Figure 3**). No classic immune related GO terms were identified, however, cell fate commitment, G-protein coupled receptor signaling, and cell–cell signaling may be immune related.

IPA identified pathways associated with genes highly expressed in the Harderian gland (**Table 2**). IPA identified more immune related pathways than the Panther GO term analysis (**Figure 3**). Notably, G-protein coupled receptor signaling was represented in both analyses (**Figure 3** and **Table 2**). Calcium Signaling, ILK signaling, CXCR4 signaling, Crosstalk between Dendritic Cells and Natural Killer Cells, and MIF Regulation of Innate Immunity are all pathways that have a direct relationship with the immune response.

**Table 2:** Canonical pathways associated with genes highly expressed in the Harderian gland.

Ingenuity canonical pathways	*p*-Value	z-Score	Genes
Calcium signaling	0.0001	2.646	20
ILK signaling	0.0005	3.771	19
Thrombin signaling	0.0008	3	19
Actin cytoskeleton signaling	0.0028	3.873	19
Protein ubiquitination pathway	0.0141	—	19
Signaling by rho family GTPases	0.0072	4	18
G-Protein coupled receptor signaling	0.0347	—	18
Tight junction signaling	0.0006	—	17
cAMP-mediated signaling	0.0112	4	17
Cardiac hypertrophy signaling	0.0186	3.207	17
Phospholipase C signaling	0.0407	3.051	16
Cellular effects of sildenafil (Viagra)	0.0003	—	15
Epithelial adherens junction signaling	0.0010	—	15
RhoGDI signaling	0.0054	-3.464	15
Agranulocyte adhesion and diapedesis	0.0129	—	15
Mitochondrial dysfunction	0.0115	—	14
Sertoli cell-Sertoli cell junction signaling	0.0158	—	14
Hepatic fibrosis/hepatic stellate cell activation	0.0195	—	14
CREB signaling in neurons	0.0214	3.162	14
CXCR4 signaling	0.0195	1.897	13
Aldosterone signaling in epithelial cells	0.0219	—	13
GABA receptor signaling	0.0001	—	11
Gαi signaling	0.0107	1.897	11
Ovarian cancer signaling	0.0363	—	11
Synaptic long term depression	0.0407	3.317	11
Corticotropin releasing hormone signaling	0.0166	3	10
RhoA signaling	0.0324	3.162	9
Oxidative phosphorylation	0.0363	—	9
Transcriptional regulatory network in embryonic stem cells	0.0002	—	8
GPCR-mediated integration of enteroendocrine signaling exemplified…	0.0102	—	8
Crosstalk between dendritic cells and natural killer cells	0.0302	—	8
Regulation of actin-based motility by rho	0.0324	2.828	8
Glutamate receptor signaling	0.0087	—	7
Agrin interactions at neuromuscular junction	0.0234	2.449	7
Remodeling of epithelial adherens junctions	0.0234	—	7
Caveolar-mediated endocytosis signaling	0.0269	—	7
Basal cell carcinoma signaling	0.0288	1.633	7
Serotonin receptor signaling	0.0079	—	6
Ethanol degradation II	0.0174	—	5
Noradrenaline and adrenaline degradation	0.0234	—	5
MIF regulation of innate immunity	0.0309	2.236	5
Triacylglycerol biosynthesis	0.0398	—	5


**FIGURE 4:**
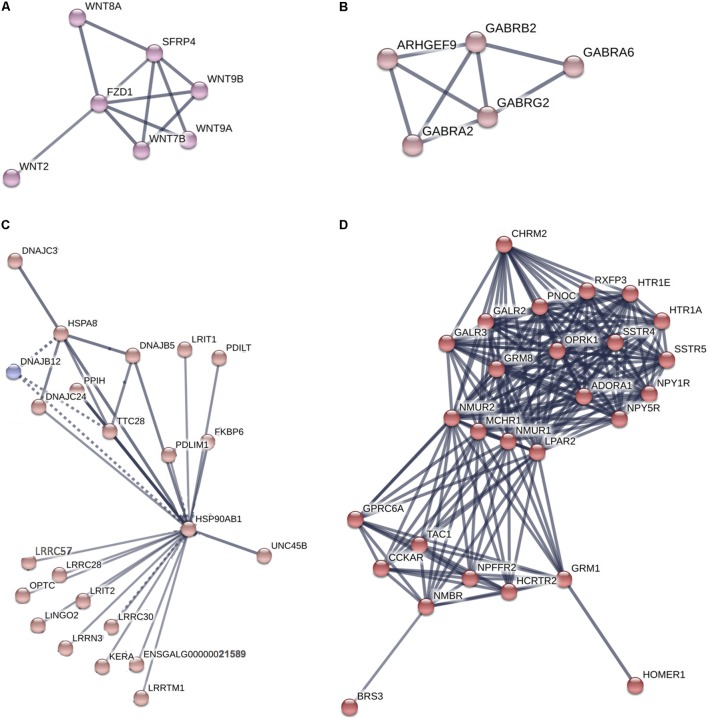
Network of genes highly expressed in the Harderian gland. Network generated by STRING. Edge thickness represents the confidence or strength of data support. A high confidence (0.700) cut-off was used to generate the network and all unconnected nodes were removed. Node color was based on MCL clustering (inflation parameter = 3). **Supplementary Figure S1** includes the full network where a total of 836 nodes and 669 edges were included. The high number of nodes and edges led to a large network. From that network four clusters of interest **(A–D)** were chosen to display.

The genes highly expressed in the Harderian gland formed a network with significantly more interactions than expected (*p* = 6.6e-16) (**Supplementary Figure S1**). STRING identified two significant (FDR < 0.05) KEGG pathways associated with these genes including Neuroactive ligand-receptor interaction (04080) and Tight junction (04530). Specific clusters of interest from the large network included Wnt genes (**Figure 4A**), GABA genes (**Figure 4B**), Heat shock proteins (**Figure 4C**), and G-protein coupled receptors (GPCR) (**Figure 4D**).

A total of 48 miRNAs were highly expressed in the Harderian gland (**Table 3**), representing 39% of the miRNAs identified in this analysis. As the library construction kit used in this study employs a poly-A-tail selection, the reads that mapped to miRNA were likely from pre-processed miRNA. The correlation between the abundance of precursor miRNA and mature miRNA is dependent on the tissue and miRNA (Lee et al., 2008). It is possible that trace amounts of mature miRNA remained after poly-A-tail selection, but because the same kit was used for all tissues analyzed in this study, we assume no bias amongst tissues. It is also possible that reads belonging to the miRNA target sequence in regulated genes were incorrectly mapped to the miRNA itself. However, since reads that mapped to multiple places in the genome were removed, this is less likely.

**Table 3:** miRNAs highly expressed in the Harderian gland.

miRNA	Accession^a^
*gga-mir-1b*	MI0001254
*gga-let-7f*	MI0001233
*gga-let-7j*	MI0001262
*gga-let-7a-1*	MI0001234
*gga-mir-29b-2*	MI0001266
*gga-mir-29c*	MI0001265
*gga-mir-30c-2*	MI0001205
*gga-mir-30d*	MI0001198
*gga-mir-34c*	MI0001261
*gga-mir-101-2*	MI0007558
*gga-mir-130a*	MI0001241
*gga-mir-130b*	MI0001239
*gga-mir-133a-2*	MI0001248
*gga-mir-133b*	MI0001206
*gga-mir-133c*	MI0001255
*gga-mir-138-2*	MI0001228
*gga-mir-144*	MI0004996
*gga-mir-193b*	MI0003698
*gga-mir-200b*	MI0001250
*gga-mir-214*	MI0008208
*gga-mir-221*	MI0001178
*gga-mir-301a*	MI0001240
*gga-mir-365b*	MI0022403
*gga-mir-365-2*	MI0003704
*gga-mir-451*	MI0004995
*gga-mir-454*	MI0006984
*gga-mir-1592*	MI0007319
*gga-mir-1640*	MI0007372
*gga-mir-1684b*	MI0022501
*gga-mir-1737*	MI0007476
*gga-mir-1764*	MI0007506
*gga-mir-1772*	MI0007515
*gga-mir-1773*	MI0007516
*gga-mir-1800*	MI0007544
*gga-mir-1812*	MI0007557
*gga-mir-2126*	MI0010731
*gga-mir-6546*	MI0022362
*gga-mir-6580*	MI0022399
*gga-mir-6606*	MI0022425
*gga-mir-6609*	MI0022428
*gga-mir-6614*	MI0022433
*gga-mir-6653*	MI0022473
*gga-mir-6663*	MI0022483
*gga-mir-6668*	MI0022488
*gga-mir-6677*	MI0022497
*gga-mir-6701*	MI0022523
*gga-mir-6704*	MI0022526
*gga-mir-7474*	MI0024147


*^*a*^Source mIRBase.*

## Discussion

The immune related genes that were highly expressed in the Harderian gland are of particular interest because they show how the homeostatic state is different compared to the bursa, spleen, and thymus. GPCR related genes, functions, and pathways were represented in every functional analysis. Every GPCR is activated by a specific ligand, which results in downstream signaling events. Glutamate is the ligand for several GPCRs identified as highly expressed in the Harderian gland, and IPA identified Glutamate Receptor Signaling as an impacted pathway. Glutamate is an amino acid that functions as a neuro-immuno-transmitter (Ganor and Levite, 2012). Glutamate can impact the immune system in several ways, i.e., affecting T cell survival, calcium levels, and cytokine expression levels (Ganor and Levite, 2012). Also, T cells, B cells, macrophages, and dendritic cells, express high levels of glutamate receptors (Ganor and Levite, 2012). The neurotransmitter GABA also acts as an immunomodulator (Jin et al., 2013). The genes highly expressed in the Harderian gland significantly impacted the GABA Receptor Signaling pathway and GABA receptors were represented in the network generated by STRING. GABA signaling can impact chemotaxis, phagocytosis, and cytokine secretion in immune cells (Jin et al., 2013). It is known there is a strong link between the central nervous system and the immune system (Black, 1994). The utilization of these neurotransmitters and their receptors in the Harderian gland compared to other immune tissues may be due to the focal role of local immunity in the Harderian gland, whereas, the other immune tissues in the current study are generally more important to systemic immunity.

Another GPCR, *FZD1*, is the receptor for Wnt proteins. Several *Wnt* genes were identified as highly expressed in the Harderian gland and were represented in the network analysis. The Wnt pathway is tightly regulated, as it is involved in development, cell differentiation, and the immune response (Staal et al., 2008). A combined heat and LPS stress event showed the bursa transcriptome decreased expression of Wnt signaling genes (Lamont, personal communication). The miRNA *mir-301a*, has been shown to be activated by the Wnt pathway in glioma cells (Yue et al., 2016). Let-7 is also involved in cell proliferation and differentiation (Roush and Slack, 2008). Previously, increased levels of *let-7* pri-miRNA were found in undifferentiated embryonic stem cells (Wulczyn et al., 2007). Increased expression of *Wnt* genes and *let-7* miRNAs in the Harderian gland may increase the efficiency of this lymphoid tissue, by serving as a home to progenitor or naïve lymphocytes that can quickly be differentiated or activated in response to a stimulus.

Small, non-coding, miRNAs regulate gene expression levels post-transcription. Many of the miRNAs identified as highly expressed in the Harderian gland influence immune related genes and pathways. In human cell lines, *mir-200b* was shown to inhibit the TLR4 pathway (Wendlandt et al., 2012), *mir-221* increased the expression of *NF*-κβ and *STAT3* (Liu et al., 2014), and *mir-193b* overexpression resulted in increased autophagy (Nyhan et al., 2016). In chickens, *mir-1764* decreased the expression levels of the inflammatory cytokine *STAT1* (Jeong et al., 2013), and *mir-30d* may regulate *IRF4* (Li et al., 2017). Avian influenza impacted the expression levels of several miRNAs in the chicken trachea and lung including the following miRNAs significant in the current study: *let-7a-1*, *let-7f*, *let-7j*, *mir-1b*, *mir-30d*, *mir-34c*, *mir-101-2*, *mir-144*, *mir-200b*, and *mir-451* (Wang et al., 2009). Increased expression of these miRNAs in the Harderian gland would clearly impact the immune response in this tissue. Using miRNAs for regulation purposes in a tissue that is required to respond quickly to pathogens that enter via the eye is a useful strategy. Since the Harderian gland transcriptome has not been analyzed until recently (Deist et al., 2018) and was not used to generate the reference genome, there were likely several Harderian gland specific lncRNAs and miRNAs not identified in this analysis. Further research is necessary to confirm the correlation of abundance between the likely precursor miRNAs identified in this study and the mature miRNA levels, and to identify tissue specific, novel RNAs.

Overall this study identified 2,386 transcripts that were highly expressed in the Harderian gland compared to the bursa, thymus, and spleen of non-challenged chickens. These transcripts highlighted the interaction between the central nervous system and the immune system via the neuro-immuno-transmitters glutamate and GABA. The Harderian gland may utilize *Wnt* genes and *let-7* miRNAs to control or stall cell differentiation. Previous studies have shown, several miRNAs identified as highly expressed in the Harderian gland have immune function. Moreover, these results elucidated the unique immune properties of the Harderian gland, a local immune tissue that must quickly respond to pathogens and vaccines that enter via the eye. It is important to gain a better understanding of the physiology of important avian tissues to develop better vaccines, breed for disease resistance in chickens, and potentially prevent zoonotic outbreaks.

## Data Availability

The datasets analyzed in this study can be found at ArrayExpress (https://www.ebi.ac.uk/arrayexpress/) or GEO (https://www.ncbi.nlm.nih.gov/geo) (see **Table 1** for more information).

## Ethics Statement

The data used in this study was derived from animal subjects, but has been downloaded from public repositories. The ethics statements can be found in the manuscripts describing the original studies (Van Goor et al., 2017; Zhang et al., 2018).

## Author Contributions

MD: collected data, processed RNA-seq data, executed analysis, data interpretation, and wrote the manuscript. SL: oversaw data collection, analysis, interpretation, read and reviewed manuscript.

## Conflict of Interest Statement

The authors declare that the research was conducted in the absence of any commercial or financial relationships that could be construed as a potential conflict of interest. The reviewer LB and handling Editor declared their shared affiliation.

## Supplementary Material

FIGURE S1 The full network from which the clusters in **Figure 4** were derived.

DATA SHEET S1 A list of the top and bottom loading genes.

DATA SHEET S2 For a complete list of the transcripts highly or lowly expressed in the Harderian gland compared to the bursa, thymus, and spleen.

## References

[B1] Albini B. Wick G. (1974). Delineation of B and T lymphoid cells in the chicken. 112 444–450. 4591972

[B2] Anders S. Pyl P. T. Huber W. (2015). HTSeq–a Python framework to work with high-throughput sequencing data. 31 166–169. 10.1093/bioinformatics/btu638 25260700 PMC4287950

[B3] Baba T. Masumoto K. Nishida S. Kajikawa T. Mitsui M. (1988). Harderian gland dependency of immunoglobulin A production in the lacrimal fluid of chicken. 65 67–71. 3181995 PMC1385021

[B4] Bailey M. J. Coon S. L. Carter D. A. Humphries A. Kim J. S. Shi Q. (2009). Night/day changes in pineal expression of >600 genes: central role of adrenergic/cAMP signaling. 284 7606–7622. 10.1074/jbc.M808394200 19103603 PMC2658055

[B5] Bang B. G. Bang F. B. (1968). Localized lymphoid tissues and plasma cells in paraocular and paranasal organ systems in chickens. 53 735–751. 5693341 PMC2013518

[B6] Black P. H. (1994). Central nervous system-immune system interactions: psychoneuroendocrinology of stress and its immune consequences. 38 1–6. 10.1128/AAC.38.1.1 8141561 PMC284388

[B7] Deist M. S. Gallardo R. A. Bunn D. A. Dekkers J. C. M. Zhou H. Lamont S. J. (2017). Resistant and susceptible chicken lines show distinctive responses to Newcastle disease virus infection in the lung transcriptome. 18:989. 10.1186/s12864-017-4380-4 29281979 PMC5745900

[B8] Deist M. S. Gallardo R. A. Bunn D. A. Kelly T. R. Dekkers J. C. M. Zhou H. (2018). Novel analysis of the Harderian gland transcriptome response to Newcastle disease virus in two inbred chicken lines. 8:6558. 10.1038/s41598-018-24830-0 29700338 PMC5920083

[B9] Fleming D. S. Koltes J. E. Fritz-Waters E. R. Rothschild M. F. Schmidt C. J. Ashwell C. M. (2016). Single nucleotide variant discovery of highly inbred Leghorn and Fayoumi chicken breeds using pooled whole genome resequencing data reveals insights into phenotype differences. 17:812. 10.1186/s12864-016-3147-7 27760519 PMC5070165

[B10] Ganor Y. Levite M. (2012). “Glutamate in the immune system: glutamate receptors in immune cells, potent effects, endogenous production and involvement in disease,” in , ed. Levite M. (Vienna: Springer), 121–161. 10.1007/978-3-7091-0888-8_4

[B11] Glick B. Chang T. S. Jaap G. (1956). The bursa of Fabricius and antibody production. 35 224–225. 10.3382/ps.0350224

[B12] Hedges S. B. (2002). The origin and evolution of model organisms. 3 838–849. 10.1038/nrg929 12415314

[B13] Huang H. Liu A. Wu H. Ansari A. R. Wang J. Huang X. (2016). Transcriptome analysis indicated that *Salmonella* lipopolysaccharide-induced thymocyte death and thymic atrophy were related to TLR4-FOS/JUN pathway in chicks. 17:322. 10.1186/s12864-016-2674-6 27142675 PMC4855877

[B14] Jeong W. Lim W. Ahn S. E. Lim C. H. Lee J. Y. Bae S. M. (2013). Recrudescence mechanisms and gene expression profile of the reproductive tracts from chickens during the molting period. 8:e76784. 10.1371/journal.pone.0076784 24098561 PMC3788108

[B15] Jin Z. Mendu S. K. Birnir B. (2013). GABA is an effective immunomodulatory molecule. 45 87–94. 10.1007/s00726-011-1193-7 22160261 PMC3680704

[B16] Kim D. Pertea G. Trapnel C. Pimental H. Kelley R. Salzberg S. L. (2013). TopHat2: accurate alignment of transcriptomes in the presence of insertions, deletions and gene fusions. 14:R13. 10.1186/gb-2013-14-4-r36 23618408 PMC4053844

[B17] Kristeen-Teo Y. W. Yeap S. K. Tan S. W. Omar A. R. Ideris A. Tan S. G. (2017). The effects of different velogenic NDV infections on the chicken bursa of Fabricius. 13:151. 10.1186/s12917-017-1071-y 28569155 PMC5452610

[B18] Lee E. J. Baek M. Gusev Y. Brackett D. J. Nuovo G. J. Schmittgen T. D. (2008). Systematic evaluation of microRNA processing patterns in tissues, cell lines, and tumors. 14 35–42. 10.1261/rna.804508 18025253 PMC2151027

[B19] Li P. Fan W. Li Q. Wang J. Liu R. Everaert N. (2017). Splenic microRNA expression profiles and integration analyses involved in host responses to *Salmonella enteritidis* infection in chickens. 7:377. 10.3389/fcimb.2017.00377 28884089 PMC5573731

[B20] Liu S. Sun X. Wang M. Hou Y. Zhan Y. Jiang Y. (2014). A microRNA 221- and 222-mediated feedback loop maintains constitutive activation of NFkappaB and STAT3 in colorectal cancer cells. 147 847.e–859.e. 10.1053/j.gastro.2014.06.006 24931456 PMC4839969

[B21] Love M. I. Huber W. Anders S. (2014). Moderated estimation of fold change and dispersion for RNA-seq data with DESeq2. 15:550. 10.1186/s13059-014-0550-8 25516281 PMC4302049

[B22] Manisikka A. Sandberg M. Veromaa T. Vainio O. Granfors K. Toivanen P. (1989). B cell maturation in the chicken Harderian gland. 142 1826–1833. 2493499

[B23] Marini F. (2016). *pcaExplorer: Interactive Visualization of RNA-seq Data Using a Principal Components Approach*. Available at: https://github.com/federicomarini/pcaExplorer.

[B24] Maslak D. M. (1994). *Head-associated Lymphoid Tissue [HALT] of the Chicken: Characterization of Lymphocytes*. Ph.D. thesis Iowa State University, Ames.

[B25] Mi H. Huang X. Muruganujan A. Tang H. Mills C. Kang D. (2017). PANTHER version 11: expanded annotation data from Gene Ontology and Reactome pathways, and data analysis tool enhancements. 45 D183–D189. 10.1093/nar/gkw1138 27899595 PMC5210595

[B26] Mueller A. P. Sato K. Glick B. (1971). The chicken lacrimal gland, gland of Harder, caecal tonsil, and accessory spleens as sources of antibody-producing cells. 2 140–152. 10.1016/0008-8749(71)90033-5 4940998

[B27] Nyhan M. J. O’Donovan T. R. Boersma A. W. Wiemer E. A. McKenna S. L. (2016). MiR-193b promotes autophagy and non-apoptotic cell death in oesophageal cancer cells. 16:101. 10.1186/s12885-016-2123-6 26878873 PMC4754993

[B28] Oláh I. Nagy N. Vervelde L. (2013). “Structure of the avian lymphoid system,” in , 2nd Edn, eds Schat K. Kaspers B. Kaiser P. (New York, NY: Elsevier Science), 456.

[B29] Ou X. Mao S. Cao J. Cheng A. Wang M. Zhu D. (2017). Comparative analysis of virus-host interactions caused by a virulent and an attenuated duck hepatitis A virus genotype 1. 12:e0178993. 10.1371/journal.pone.0178993 28614378 PMC5470708

[B30] Pritchett E. M. Lamont S. J. Schmidt C. J. (2017). Transcriptomic changes throughout post-hatch development in *Gallus gallus* pituitary. 58 43–55. 10.1530/JME-16-0186 27856505 PMC5148799

[B31] Roush S. Slack F. J. (2008). The let-7 family of microRNAs. 18 505–516. 10.1016/j.tcb.2008.07.007 18774294

[B32] Russell P. H. Dwivedi P. N. Davison T. F. (1997). The effects of cyclosporin A and cyclophosphamide on the populations of B and T cells and virus in the Harderian gland of chickens vaccinated with the Hitchner B1 strain of Newcastle disease virus. 60 171–185. 10.1016/S0165-2427(97)00094-9 9533275

[B33] Sandford E. E. Orr M. Balfanz E. Bowerman N. Li X. Zhou H. (2011). Spleen transcriptome response to infection with avian pathogenic *Escherichia coli* in broiler chickens. 12:469. 10.1186/1471-2164-12-469 21951686 PMC3190404

[B34] Shoemaker J. E. Lopes T. J. Ghosh S. Matsuoka Y. Kawaoka Y. Kitano H. (2012). CTen: a web-based platform for identifying enriched cell types from heterogeneous microarray data. 13:460. 10.1186/1471-2164-13-460 22953731 PMC3473317

[B35] Smith A. L. Göbel T. W. (2013). “Avian T cells: antigen recognition and lineages,” in , 2nd Edn, eds Schat K. Kaspers B. Kaiser P. (New York, NY: Elsevier Science), 456.

[B36] Staal F. J. Luis T. C. Tiemessen M. M. (2008). WNT signalling in the immune system: WNT is spreading its wings. 8 581–593. 10.1038/nri2360 18617885

[B37] Sun H. Liu P. Nolan L. K. Lamont S. J. (2015). Novel pathways revealed in bursa of Fabricius transcriptome in response to extraintestinal pathogenic *Escherichia coli* (ExPEC) infection. 10:e0142570. 10.1371/journal.pone.0142570 26556806 PMC4640532

[B38] Sun H. Liu P. Nolan L. K. Lamont S. J. (2016). Thymus transcriptome reveals novel pathways in response to avian pathogenic *Escherichia coli* infection. 95 2803–2814. 10.3382/ps/pew202 27466434 PMC5144662

[B39] Szklarczyk D. Morris J. H. Cook H. Kuhn M. Wyder S. Simonovic M. (2017). The STRING database in 2017: quality-controlled protein-protein association networks, made broadly accessible. 45 D362–D368. 10.1093/nar/gkw937 27924014 PMC5210637

[B40] Van Goor A. Ashwell C. M. Persia M. E. Rothschild M. F. Schmidt C. J. Lamont S. J. (2017). Unique genetic responses revealed in RNA-seq of the spleen of chickens stimulated with lipopolysaccharide and short-term heat. 12:e0171414. 10.1371/journal.pone.0171414 28166270 PMC5293231

[B41] Wang Y. Brahmakshatriya V. Zhu H. Lupiani B. Reddy S. M. Yoon B. J. (2009). Identification of differentially expressed miRNAs in chicken lung and trachea with avian influenza virus infection by a deep sequencing approach. 10:512. 10.1186/1471-2164-10-512 19891781 PMC2777939

[B42] Wendlandt E. B. Graff J. W. Gioannini T. L. McCaffrey A. P. Wilson M. E. (2012). The role of microRNAs miR-200b and miR-200c in TLR4 signaling and NF-kappaB activation. 18 846–855. 10.1177/1753425912443903 22522429 PMC3733339

[B43] Wight P. A. Burns R. B. Rothwell B. Mackenzie G. M. (1971). The Harderian gland of the domestic fowl I. Histology, with reference to the genesis of plasma cells and Russell bodies. 110 307–315. 4111364 PMC1271098

[B44] Wulczyn F. G. Smirnova L. Rybak A. Brandt C. Kwidzinski E. Ninnemann O. (2007). Post-transcriptional regulation of the let-7 microRNA during neural cell specification. 21 415–426. 10.1096/fj.06-6130com 17167072

[B45] Yue X. Cao D. Lan F. Pan Q. Xia T. Yu H. (2016). MiR-301a is activated by the Wnt/beta-catenin pathway and promotes glioma cell invasion by suppressing SEPT7. 18 1288–1296. 10.1093/neuonc/now044 27006177 PMC4998999

[B46] Zhang J. Kaiser M. G. Deist M. S. Gallardo R. A. Bunn D. A. Kelly T. R. (2018). Transcriptome analysis in spleen reveals differential regulation of response to newcastle disease virus in two chicken lines. 8:1278. 10.1038/s41598-018-19754-8 29352240 PMC5775430

