# Supplementary figures and images for: What Makes the Harderian Gland Transcriptome Different From Other Chicken Immune Tissues? A Gene Expression Comparative Analysis
Source: Front Physiol. 2018 May 8;9:492. doi: 10.3389/fphys.2018.00492 (PMC5952037; doi:10.3389/fphys.2018.00492)

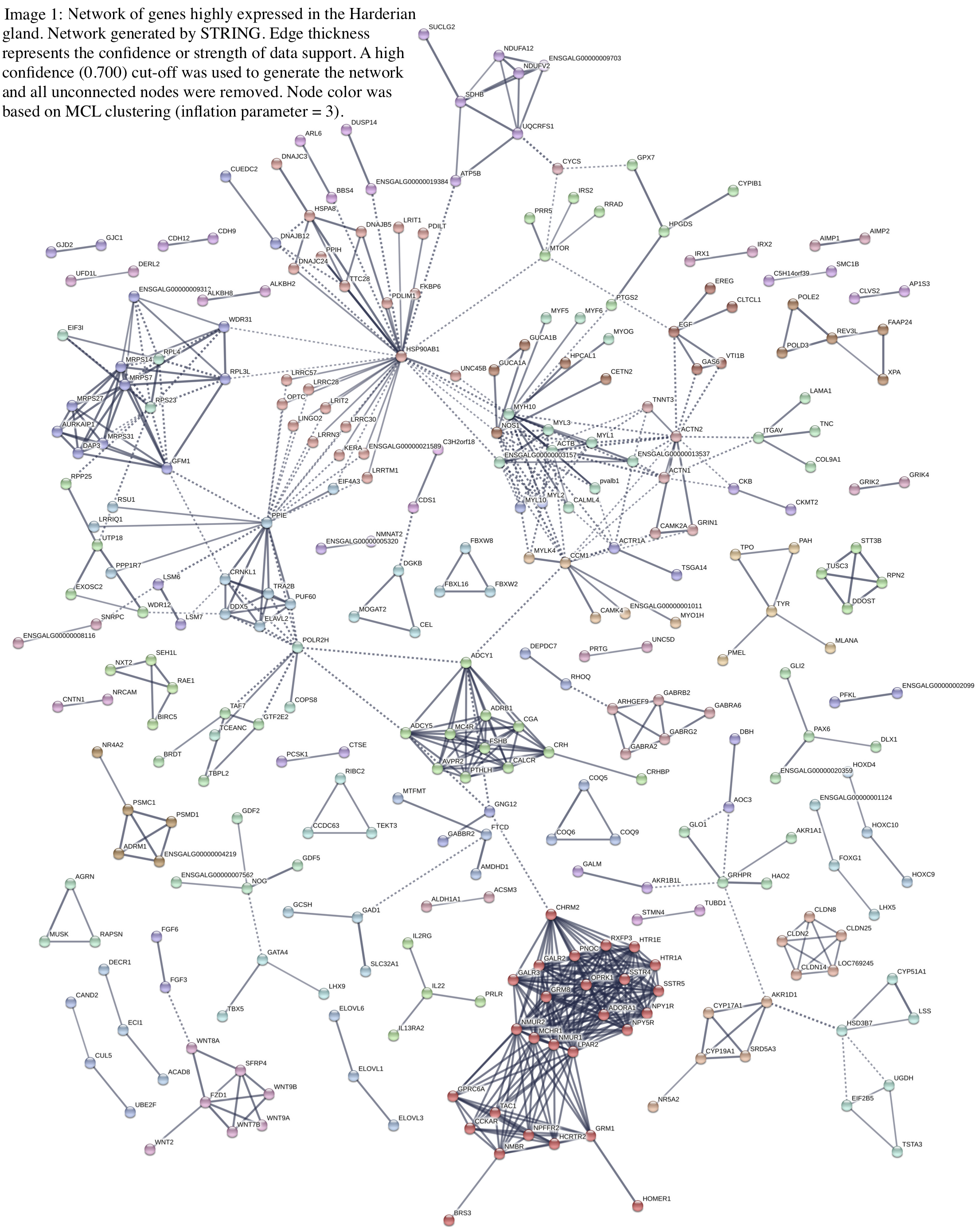

Supplement: FIGURE S1 — The full network from which the clusters in Figure 4 were derived. [file Image_1.TIFF]
